# Evaluation of a Precision Biotic on the Growth Performance, Welfare Indicators, Ammonia Output, and Litter Quality of Broiler Chickens

**DOI:** 10.3390/ani12030231

**Published:** 2022-01-19

**Authors:** Vincent Jacquier, Maria Carol Walsh, Ghislain Schyns, Joshua Claypool, Britt Blokker, Cristiano Bortoluzzi, Jack Geremia

**Affiliations:** 1 DSM Nutritional Products, 68128 Village-Neuf, France; vincent.jacquier@hotmail.fr (V.J.); Ghislain.schyns@dsm.com (G.S.); britt.blokker@dsm.com (B.B.); 2 DSM Nutritional Products, 4303 Kaiseraugst, Switzerland; Maria.Walsh@dsm.com; 3 DSM Nutritional Products, Lexington, MA 02421, USA; joshua.claypool@dsm.com (J.C.); jack.geremia@dsm.com (J.G.)

**Keywords:** broiler, footpad dermatitis, microbiome metabolic modulator, sustainability

## Abstract

**Simple Summary:**

A novel precision biotic was tested to evaluate its impact on growth performance, welfare and ammonia output and litter quality in broiler chickens. The supplementation improved broiler performance, which was found to be at least partially related to reductions in footpad lesions. These improvements appeared to be mediated through improvements in the litter quality, reductions in litter pH, and reductions in ammonia concentrations in litter.

**Abstract:**

A dietary glycan-based precision biotic (Glycan PB) was evaluated on the performance, welfare indicators, and litter characteristics of broiler chickens. In Trial 1, the main effects of Glycan PB dose (0, 250 and 500 g/metric ton (MT)) and xylanase supplementation (0 or 100 g/MT) were tested, as was their interaction. In Trial 2, pens located inside a commercial house were used to test the effect of Glycan PB supplementation (500 g/MT) versus a control diet. In Trial 1, Glycan PB supplementation at 250 and 500 g/MT improved feed conversion ratio (FCR) by 7 and 11 points when compared to diets without Glycan PB (*p* < 0.001). At 35 d, Glycan PB reduced the pH and ammonia concentration in diets with xylanase. In Trial 1, the supplementation with 500 g of Glycan PB/MT of feed reduced litter scores (*p* < 0.05). In both trials, 500 g of Glycan PB/MT of feed increased the proportions of birds without footpad lesions (Trial 1: 72.2% vs. 82.7%; *p* < 0.001; Trial 2: 14 to 27.3% (*p* = 0.05) or gait defects (Trial 1: 96.1% vs. 98.4%; *p* < 0.001) and decreased the proportion of birds with footpad lesions (Trial 2: 86% vs. 72.7%; *p* = 0.05).

## 1. Introduction

Global efforts to reduce antibiotic use in the poultry industry have gathered momentum over the last decade, prompting the development of new technologies to improve animal nutritional health, performance, welfare, and sustainability. In parallel, significant advances in molecular and computational biology have also provided a breakthrough in the scientific understanding of the gut microbiome and its metabolic functions across a variety of animal species [1,2]. 

Precision biotics (PB) are a new category of nutritional feed ingredients being developed to leverage the recent advances in microbiome science to answer new challenges in the poultry and other protein production industries [3,4,5]. The mode of action employed by PB is unlike that of probiotics, prebiotics or other conventional gut-health products. Precision biotics are microbiome metabolic modulators (MMM) that influence selected metagenomic functions of the gut microbiome, thereby modulating the production of microbial metabolites targeted specifically to promote beneficial outcomes to the animal and the environment [3,4,5]. Unlike existing technologies that modulate the microbiota, the primary action of PB is through the modulation of the abundance of metabolic pathways rather than the modulation of bacterial taxa, i.e., gut microbial profiles [5].

The reasoning behind PB is to be able to target metagenomic functions, based on a desire to provide a more consistent response than would normally be obtained by using conventional gut health products that target microbial taxonomic abundance. It has been shown conclusively, in humans and other hosts, that there is far less variability in microbiome metabolic function across individuals than there is in their underlying taxonomic composition [6]. In other words, the aggregated pathways for core metabolic functions of the microbiome [7] are often strongly conserved across individuals, even when the relative abundance of different microbial species varies significantly.

Precision biotics are carbohydrates with glycosidic linkages and size distributions selected specifically for their ability to modulate microbiome pathways such as short-chain fatty acid production and amino acid metabolism in the gut [5]. For example, it has been found that a specifically selected PB product (Glycan M2-1, Midori USA, Inc., Cambridge, MA, USA, DSM Nutritional Products, Kaiseraugst, Switzerland) modulated the abundance of genes found in microbial pathways associated with propionate production and amino acid metabolism, including pathways that improve energy efficiency [8,9] and reduced intestinal ammonia production, with an improvement observed in growth performance [5].

Footpad dermatitis is characterized by necrotic lesions on the footpads [10], is an important indicator of welfare in broiler chickens and is known to be directly affected by husbandry conditions and farm management [11], and indirectly affected by nutritional management. Additionally, ammonia emissions arising from animal production are under scrutiny due to their negative impact on air quality, eutrophication, and acidification, affecting human health and the health of many terrestrial and aquatic ecosystems [12]. Therefore, we hypothesized that the modulation of microbial short-chain fatty acids (SCFA) and amino acid metabolic pathways by Glycan PB would translate into an in vivo reduction of intestinal ammonia and improved litter quality, thus resulting in better animal welfare (e.g., reductions in footpad dermatitis) and reduced environmental emissions from the litter.

The objective of this work was to evaluate the effects of a Glycan-based PB on the performance, footpad lesions, locomotion, and litter characteristics of broiler chickens. The PB was selected for its ability to activate the propionic acid (C3) and butyric acid (C4) bio-synthesis pathways, and to modulate amino acid degradation and amine biosynthesis. In the first trial, the optimal dose of PB and its interaction with dietary xylanase supplementation were also evaluated. The second trial was conducted with commercial-type pens, with an aim to replicate the effects in a subset of end points.

## 2. Materials and Methods

### 2.1. Animal Ethics

Two animal trials were designed and conducted in 2019 in accordance with the requirements of Directive 2010/63/EU on the protection of animals used for scientific purposes. Protocols were subject to review and approval by Company Animal Experimentation Committees (Agrisearch, Pécel, Hungary; CTPA, Ploufragan, France).

### 2.2. Trial 1

A total of 2340 one-day-old male Ross 308 broiler chickens were obtained from a commercial hatchery and allocated into 78 floor pens with 30 birds per 2.70 m² pen. Unhealthy chicks (leg problem, unhealed navel) were removed from the study. Healthy chicks were allocated into pens according to their initial body weight (41.4 ± 0.1 g). Each pen was uniquely identified. Re-used wood shaving litter from clinically healthy and unmedicated birds was used. The re-used litter was homogeneously mixed and equally distributed among the pens. Feed and water were provided *ad libitum* by bell feeders and drinkers throughout the experimental period until d 35.

Environmental temperature was 33 °C during the first week and reduced by 3 °C per week until 21 °C, where it was maintained until the end of the trial at 35 d. The lighting program gradually increased the hours of darkness from 0 h at placement to 6 h at d 6. After d 28, the hours of darkness were gradually reduced to 1 h of darkness at d 35. The ventilation followed the breeder guidelines.

### 2.3. Trial 2

A total of 450 one-day-old male Ross 308 broilers were allocated into 30 floor pens located in one commercial broiler house, with 15 broilers per pen. The total area of each pen was 1 m², with 0.87 m² of usable surface. After disinfection of the buildings and before the arrival of the chickens, new, clean straw pellets were added to the pens to act as bedding material. The chicks were sourced from a local hatchery (Couvoir Josset, Caro, France). One day after arrival, the chicks were weighed, identified, and distributed into floor pens according to their initial body weight (51.1 ± 0.4 g). Environmental temperature was 33 °C during the first week and reduced 3 °C per week until 21 °C, which was maintained until the end of the trial at 35 d. The lighting program gradually increased from 0 h of darkness at placement, to 6 h at d 6. After d 28, darkness hours were gradually reduced to 1 h of darkness at d 35. The ventilation regime followed the breeder guidelines.

### 2.4. Experimental Diets

Experimental diets for both trials were provided as crumbles in the starter, and pellets in grower and finisher diets, and were designed to meet or exceed the nutritional requirements specified by the primary breeder (Aviagen Inc., Huntsville, AL, USA). Temperature during pelleting did not exceed 65 °C. Diet compositions and specifications for both trials are shown in Table 1. In Trial 1, the feeding program consisted of: starter diet from 0 to 14 days, grower diet from 14 to 28 days, and finisher diet from 28 to 35 days. Trial 1 used wheat-soybean meal-based diets with an inclusion of barley (<8%). In Trial 2, the feeding program consisted of: starter diet from 0 to 10 days, grower diet from 10 to 28 days, and finisher diet from 28 to 35 days. Trial 2 used a diet based on corn, wheat, and soybean meal, and used a non-starch polysaccharide-hydrolyzing enzyme (1500 viscometric units of endo-1,3(4)-ß-glucanase and 1100 viscometric units of endo-1,4-ß-xylanase, Rovabio^®^ Excel AP, Adisseo). All diets contained phytase (1000 FYT/kg, RONOZYME^®^ HiPhos, DSM Nutritional Products).

### 2.5. Experimental Design

Trial 1 had a completely randomized block design with a factorial arrangement of treatments, testing the main effects of Glycan PB dose (0, 250 and 500 g/metric ton (MT), and xylanase supplementation (diets with 100 g/MT or without RONOZYME^®^ WX 2000 CT, DSM Nutritional Products, Switzerland), as well as their interaction. The blocks were intended to capture spatial variations within the barn. Trial 2 had a completely randomized design and tested the effect of Glycan PB supplementation using a control diet, and the control diet plus supplementation with Glycan PB at 500 g/MT.

The study variables included BW and cumulative feed intake (FI) at each time of diet change (d 14 and d 28 in Trial 1; d 10 and d 28 in Trial 2), as well as at the end of the trial at d 35. The feed conversion ratio (FCR) was calculated as the pen total feed intake divided by the pen total weight gain. FCR was mortality-adjusted by adding back the weight of dead birds to the total pen weight and then correcting to a common weight (cFCR) using a correction coefficient calculated from published growth data for the corresponding bird genetics. Mortality was recorded during the entire experimental period. Trial 1 also evaluated litter pH, total and inorganic litter N, ammonia concentration in litter, litter scores, footpad lesion scores, and gait scores. In Trial 2, the intra-pen coefficients of variation in BW and footpad lesion scores were also evaluated.

### 2.6. Footpad Lesions and Gait Score

In Trial 1, footpad lesions and gait scores were evaluated for each bird within a pen by direct observation at d 35, whilst in Trial 2, only footpad lesions were evaluated (Table 2). For the statistical analysis, differences in the proportions of birds within a pen with score 0 (no signs of footpad dermatitis or normal gait with no detectable abnormalities), and scores greater than 0 were analyzed.

### 2.7. Litter Characteristics

In Trial 1, triplicate suspensions of 5.0 g of poultry litter were used to determine the pH. A total of 150.0 g of deionized water was added to each sample and the pH was measured by portable HI 99163 pH-meter (Hanna Instruments, Vöhringen, Germany). Litter quality was assessed immediately after the birds had been removed from the house by three independent observers on d 35. The scoring system is described in Table 2 [13]. At 35 d, litter samples were collected at four locations in each pen, avoiding the drinking area. These four samples were homogenized to form one composite sample per pen, which was then subsampled. Litter samples were stored at −20 °C immediately post-collection. The ammonia concentration in litter, total N, and inorganic N were analyzed according to standard methods [14]. The ammonia in the litter was determined by direct nesslerization using a DR2800 spectrophotometer (HACH Company, Loveland, CO, USA). Nitrogen concentrations were determined using a Kjeldahltherm microsystem, turbosog equipped (C. Gerhardt GmbH & Co. KG, Königswinter, Germany) and a Vadopest 200 steam distillation unit (C. Gerhardt GmbH & Co. KG, Königswinter, Germany). Ammonia, total and inorganic N are presented in mg/g of dry litter. The proportions of ammonia to inorganic and total N concentrations were calculated and analyzed.

### 2.8. Precision Biotic (Glycan-Based Precisio Biotic)

A glycan feed ingredient was produced by the catalytic oligomerization of food sugars into tailored glycans, as described previously [3,4]. Structural characterization of glycans was performed using the methods as described by Geremia et al. [15]. Glycan PB (Midori USA, Inc., Cambridge MA, DSM Nutritional Products, Kaiseraugst, Switzerland) was prepared at 1 kg scale from food-grade glucose monohydrate (CAS no. 14431-43-7, Sigma-Aldrich, St. Louis, USA). The number-average and weight-average molecular weights of the resulting gluco-oligosaccharides were determined by size exclusion chromatography (HPLC/SEC) to be Mn = 773 ± 37 g/mol and Mw = 1181 ± 90 g/mol, respectively.

The glycosidic linkage distribution of Glycan PB was characterized by two-dimensional heteronuclear single quantum coherence nuclear magnetic resonance spectroscopy (2D 1H13C HSQC NMR). Spectra were analyzed using MestReNova version 11.0.4-18998 (Mestrelab Research S.L., Santiago de Compostela, Spain) to determine the relative abundance for peaks within the anomeric region of the 2D NMR spectrum. Identifying peaks were characterized as follows: (d1 = 103.39 ppm, d2 = 4.50 ppm) 20.8% ± 0.4%, (d1 = 98.50 ppm, d2 = 4.95 ppm) 30.1 % ± 0.8%, (d1 = 99.71 ppm, d2 = 5.34 ppm) 8.6% ± 0.2%, (d1 = 100.25 ppm, d2 = 5.39 ppm) 4.9% ± 0.1%, (d1 = 104.54 ppm, d2 = 4.62 ppm) 2.8% ± 0.1%.

Glycan PB presented in a syrup form with a concentration of 60% Glycan PB was mixed to the feed. Before feed application, 1333 mL of this syrup was diluted in 667 mL of distilled water at 40 °C to achieve 2000 mL. The syrup was added to 3 kg of the base diet mix and agitated until all the syrup was absorbed. The flowable syrup-coated material was then added to the feed during manufacture. Doses of 250 and 500 g/MT feed refer to the concentrations of syrup solids in the feed.

### 2.9. Statistical Analysis

The data were quality checked for normality using the Fligner–Killeen test for homogeneity of variances. No outliers were excluded from the data set due to statistical criteria. In trial 1, linear mixed-effect models were constructed for cumulative feed intake, BW gain, and cFCR, with xylanase, Glycan PB dose and their interaction as the fixed effect and with blocks as random, using the lme function from the nlme package of R [16]. Non-normal data for footpad lesion scores, gait scores, and ratios of ammonia to total and inorganic N were analyzed using the non-parametric raov function of the Rfit package of R [17]. In trial 2, linear models were constructed for cumulative feed intake, BW gain, cFCR, CV of BW, and footpad lesion scores with the inclusion of Glycan PB as fixed effect, using the lme function of R [18]. Pair-wise comparisons with a Tukey’s adjustment were performed using the same models as the multicomp package of R [19]. Significance was assessed with an alpha probability of 0.05.

## 3. Results

### 3.1. Growth Performance

#### 3.1.1. Trial 1

Table 3 and Table 4 show the effects of xylanase, Glycan PB dose and their interaction on broiler performance and litter, footpad health, and gait characteristics from Trial 1. No statistically significant treatment effect was observed for mortality. Feed intake was significantly decreased by Glycan PB at 0–14 d (*p* < 0.01) and 0–35 d (*p* < 0.001), with no significant effects of xylanase and no interactions between xylanase and Glycan PB dose. In the 0–14 d period, birds receiving 250 g/MT of Glycan PB showed decreased FI compared to birds without Glycan PB supplementation, whereas in the 0–35 d period, both 250 g/MT and 500 g/MT levels decreased FI. No effects of xylanase or Glycan PB on FI were observed from d 0–28. No significant interaction between xylanase and Glycan PB dose were observed for BW gain. However, BW gain was increased by both 250 and 500 g Glycan PB /MT feed in the overall period, by 40 and 42 g/bird, respectively (*p* < 0.001), which was also observed in the 0–14 and 0–28 d periods, without the effects of xylanase.

No interaction between xylanase and Glycan PB was observed on the cFCR in any of the periods evaluated (*p* > 0.05). Significant effects of Glycan PB dose on the cFCR were detected during the experimental period. From 0–14 d, 250 and 500 g Glycan PB /MT feed improved the cFCR compared to diets without Glycan PB supplementation (*p* < 0.001), without differences between the two doses of Glycan PB. The dose of 250 g Glycan PB /MT improved the cFCR by 5 points on d 28, and by 7 points on d 35, as compared to diets without Glycan PB. A dose of 500 g Glycan PB/MT feed improved the cFCR by 7 points on d 28 (*p* < 0.001), and by 11 points on d 35 (*p* < 0.001) compared to diets without Glycan PB. Additionally, significant differences were seen between the 250 and 500 g Glycan PB /MT feed levels for cFCR on d 28 and 35.

#### 3.1.2. Trial 2

Table 5 shows the effects of Glycan PB supplementation on broiler performance, BW variability, and footpad health from Trial 2. Significant effects of Glycan PB supplementation on FI were observed during 0–25 d and 0–35 d (*p* = 0.04 and *p* = 0.03, respectively), with an increased FI in birds supplemented with Glycan PB. No significant differences were seen for BW gain or cFCR as a result of dietary Glycan PB supplementation. However, Glycan PB tended to increase BW gain by 112 g/bird (*p* = 0.10) over the entire experimental period, and no significant differences in the CVs of BW were detected in Trial 2.

### 3.2. Litter Characteristics, Footpad, and Gait Score

In Trial 1 (Table 4), a significant interaction between Glycan PB and xylanase was observed for litter pH (*p* = 0.04), inorganic N (*p* = 0.001), and ammonia (*p* = 0.001) concentrations in litter. The interaction demonstrated that the effect of Glycan PB dose on litter pH was stronger for diets that contained xylanase, resulting in a reduction of litter pH from 9.80 to 8.64 in those diets, when supplemented with 500 g Glycan PB/MT feed. Similarly, the concentration of inorganic N and ammonia in litter was reduced in response to Glycan PB supplementation only in the diets containing xylanase. In those diets, ammonia concentration was reduced from 31.45 mg/g to 10.50 and 14.15 mg/kg of ammonia with 250 and 500 g Glycan PB/MT feed, respectively. A main effect of Glycan PB dose on inorganic N and ammonia concentrations in litter indicated a reduction in response to 250 g Glycan PB/MT.

The ratio between ammonia and inorganic N concentrations in the litter showed an interaction (*p* < 0.001) between Glycan PB and xylanase, where 250 g Glycan PB/MT reduced the ratio by 6.1 points in diets containing xylanase. A similar interaction (*p* < 0.001) between Glycan PB and xylanase was observed for the ratio of ammonia to total N concentration in litter, i.e., in the diets containing xylanase only, Glycan PB reduced the ammonia to total N concentration in litter from 67% to 17% and 26% for 250 and 500 g Glycan PB /MT, respectively (Table 4). 

Litter scores at d 35 were decreased by 500 g Glycan PB/MT feed compared to diets without Glycan PB (Table 4; *p =* 0.05). In Trial 1, the proportion of birds without footpad lesions was affected by the Glycan PB dose, and an interaction with xylanase was recorded (*p* = 0.05). A dose of 500 g Glycan PB/MT feed without xylanase showed a significant increase in the proportion of birds with no signs of footpad lesions (score of 0) from 70.2% to 83.9%. When xylanase was added to the feed, Glycan PB numerically increased the proportion of birds without lesions. Gait scores from Trial 1 showed a main effect for Glycan PB dose (*p* = 0.03). A dose of 250 g Glycan PB/MT feed was sufficient to increase the proportion of birds without locomotory problems (gait score 0) from 96.1 to 98.4% compared to non-supplemented birds, and a dose of 500 g Glycan PB/MT feed was similarly effective, and both doses of Glycan PB significantly reduced (*p* = 0.03) the proportion of birds with gait scores between 1 and 5 (Table 4). 

In Trial 2, a numerical reduction in footpad score in response to Glycan PB was observed (*p* = 0.10; Table 5), which was confirmed with a significant increase in the proportion of birds without any signs of footpad dermatitis from 14 to 27.3% (*p* = 0.05), and a reduction of birds with scores between 1 and 4 from 86 to 72.7% (*p* = 0.05).

## 4. Discussion

This study evaluated the effects of a Glycan-based PB selected for its ability to activate the C3 and C4 SCFA bio-synthesis pathways and modulate amino acid degradation and amine biosynthesis [5], on the performance, footpad lesions, locomotion, and litter characteristics of broiler chickens. With that aim, two trials were performed, one in a controlled experimental facility (Trial 1) and one using portable divisions within a commercial broiler facility (Trial 2) to reflect the environmental conditions in the field. The first trial assessed the optimal dose of Glycan PB and its interactions with dietary xylanase, with chickens fed a wheat-based diet that contained barley to increase the sensitivity of the model in terms of litter quality and footpad lesions. In the second trial, a simplified approach to suit commercial conditions used only one Glycan PB dose and a commercial wheat/corn-based diet.

The performance response of broilers in the first experiment was clearly affected by the dietary inclusion of Glycan PB, with the low dose (250 g/MT) being sufficient to elicit an effect on FI and BW gain, but the higher dose (500 g/M) showing additional benefits in terms of cFCR. Although Glycan PB did not significantly affect BW gain and cFCR in the second experiment, there was a trend (*p* = 0.10) towards an increase in BW gain by 112 g in in response to Glycan PB supplementation, and a numeral improvement in cFCR (4 points; *p* = 0.42). Clearly, the greater level of variation under commercial conditions affected our ability to detect differences in the second trial.

One relevant finding from this study was an overall lack of interaction between Glycan PB and xylanase on broiler performance. Dietary xylanase has been reported to induce increments in SCFA production in the caeca of broilers fed wheat- and corn-based diets, driven mainly by changes in the acetic acid production [20], increments in acetic and butyric acids [21], or lack of effects on SCFA concentrations in the caeca of broilers [22]. The current results suggest that the mechanisms by which the Glycan PB ingredient elicited responses affecting broiler performance were independent from those of xylanase. There is not enough evidence in this study to attribute those independent mechanisms to the effects of Glycan PB on specific microbiome pathways. However, recent data suggest a marked effect of Glycan PB on upregulating the propionic acid metabolic pathways and amino acid metabolism pathways of the chicken cecal microbiome in vivo [5], which might differentiate it from any effects of xylanase.

Interestingly, the optimal dose of Glycan PB to elicit an improvement in cFCR was lower in the starter diet (250 g/MT), as compared to the grower or finisher diets (500 g/MT). This observation would suggest differences in the response of the microbiota based on age. For instance, it is possible that bacterial succession in the intestine of chickens [23] determines the response to Glycan PB, or that such succession is influenced by Glycan PB, which requires further study.

Remarkably, litter pH showed a clear interaction between Glycan PB dose and xylanase inclusion in Trial 1. Xylanase inclusion has been reported to have negligible effects on litter pH in other studies, even when barley was included in the diet [24]. Cengiz et al. [24] also reported no effects of xylanase on footpad lesions, even when the problem was exacerbated with increased litter moisture. Even though neither xylanase inclusion nor Glycan PB dose had effects on total N concentration in litter, there were interactions for both inorganic N and ammonia, which are highly correlated, as most of the inorganic N in litter is in the form of ammonia. Similar to the effects on pH, the effects of Glycan PB on ammonia and inorganic N concentrations were observed in diets with xylanase, which, in this particular diet, indicated increased ammonia concentrations when xylanase was present. Such increments in ammonia concentrations were reduced by Glycan PB supplementation. Overall, 250 g/MT reduced ammonia concentration in litter by 51% compared to diets without Glycan PB, demonstrating the potential of this technology to reduce ammonia output to the environment and suggesting a non-linear dose response.

Uric acid is the primary source of N in poultry litter, which is quickly converted to urea and then hydrolyzed into NH_3_ in the litter environment. Ammonia is partitioned into solid, liquid and gas phases depending on the conditions of the environment [25]. The differences in the proportion of ammonia to total N and inorganic N, which were reduced by Glycan PB when xylanase was present, suggest that the process of conversion of uric acid and urea to ammonia was delayed, or that N was diverted towards amino acid biosynthesis or amine production due to the presence of the Glycan PB either inside the animal or in the litter. Walsh et al. [5] recently observed that the cecal microbiome of broilers had an increased abundance of the arginine-N-succinyl transferase gene in response to dietary Glycan PB supplementation, suggesting that the flow of nitrogen in the intestinal lumen may be at least partially redirected away from ammonia into glutamate in the animal. The present study suggests that such changes in fermentation in response to Glycan PB are likely modified by xylanase, which solubilizes oligo-arabinoxylans and may promote changes not only to the abundance of prebiotic bacterial species [21], but also to their metabolic outputs. Nonetheless, the biochemical nature of the interaction between xylanase and Glycan PB on ammonia production is still to be elucidated.

The interactions between Glycan PB dose and xylanase on footpad health showed a slightly different pattern compared to the interaction for litter pH or ammonia concentrations in litter, demonstrating a greater effect (lower pH and lower ammonia concentration) of Glycan PB in diets without xylanase. Nevertheless, the main effect of Glycan PB on footpad health and gait ability of the birds indicates a potential for this ingredient to positively affect chicken welfare. There is a large body of research showing a relationship between poor litter quality, in particular high litter moisture, and greater prevalence of footpad dermatitis in chickens [10], which has also been associated with reduced locomotion as measured by gait scores [26]. Equally, there is a clear causal link between litter pH and the prevalence of footpad lesions in broilers, where litter changes by reducing pH can also reduce the presence of footpad lesions [27].

Additionally, it is known that higher litter pH increases the volatilization of ammonia from litter, in particular, when pH is greater than 7 [28], and high moisture and high temperatures are present [29]. Therefore, the concomitant presence of high litter moisture, high temperature, and high pH creates ideal conditions for an increased conversion of uric acid and urea to ammoniacal nitrogen [25], increasing the presence of footpad lesions, and ammonia volatilization, producing respiratory and mucosal irritation in the birds [30], ultimately decreasing broiler performance and economic returns. For instance, in the study reported by De Jong et al. [26] in semi-commercial facilities, an induced increment in litter moisture that decreased the presence of birds with footpad lesion scores from 50–70% to 0–5% produced a simulated financial gross margin reduction of approximately 40%.

In the current study, supplementation of Glycan PB at 500 g/MT decreased pH by log_10_ 0.8, improved the observed litter quality by 0.4 points, increased numbers of birds with no sign of footpad lesions from 72.2 to 82.7%, and increased the presence of birds with gait scores of 0 from 96.1 to 98.4. Although the incidence and severity of footpad lesions and locomotory problems was not severe, improving litter quality can at least partially be responsible for the observed effects in broiler performance. 

The second trial, which was carried out in a commercial environment and exhibited greater variability, supported the findings of Trial 1 with a trend towards a reduction of footpad lesion scores of 0.6 points, and an increase in the proportion of birds with no signs of footpad lesions from 14.0% to 27.3%. In this case, the footpad lesion scores of the control birds were significantly higher compared to Trial 1 (2.75 versus 0.40 points in Trial 1), suggesting a greater challenge for all the birds in Trial 2 that might have contributed to this level of variation and a poor overall performance. Interestingly, under these conditions, the main effect of Glycan PB on performance was by increasing FI by 164 g feed/bird, accompanied by an increasing trend in BW gain by 112 g. It is possible that the severity of the challenges on foot health and locomotion may have created a greater potential for the additive, as opposed to feed efficiency, to improve intake and growth.

## 5. Conclusions

Overall, the results presented herein indicate that the supplementation of 500 g/MT of Glycan PB improved broiler performance, which was found to be at least partially related to reductions in footpad lesions. These improvements appeared to be mediated through improvements to the litter quality and reductions in litter pH, and reductions to ammonia concentrations in litter. Further studies are required to determine the extent to which welfare effects can reflect the reduced ammonia concentrations in the litter versus changes in the production of SCFAs due to the microbiome metabolic modulator used herein. Microbiome metabolic modulators have the potential to modulate the functionality of the gut microbiome by specifically improving the efficiency of microbial carbon and nitrogen utilization, enhancing the sustainability of broiler production by improving broiler welfare and reducing ammonia output to the environment while improving the productivity of the poultry production system.

## Figures and Tables

**Table 1:** Ingredient composition and nutritional specifications of experimental diets of two pen trials with broiler chickens.

Ingredients and Specifications		Trial 1			Trial 2		
		Starter (0–14 d)	Grower (14–28 d)	Finisher (28–35 d)	Starter (0–10 d)	Grower (10–28 d)	Finisher (28–35 d)
Corn	kg/MT	0.0	0.0	0.0	239.4	189.4	149.4
Wheat	kg/MT	560.0	560.0	600.0	368.1	478.3	529.5
Soybean meal	kg/MT	240.0	230.0	190.0	332.6	266.3	231.8
Barley	kg/MT	50.0	85.0	78.5	0.0	0.0	0.0
Full-fat soy	kg/MT	90.0	60.0	60.0	0.0	0.0	0.0
Soy oil	kg/MT	18.0	24.0	30.0	11.7	25.6	52.9
Limestone	kg/MT	12.5	12.5	12.0	9.1	8.1	8.2
MCP	kg/MT	12.5	10.5	12.5	0.0	0.0	0.0
DCP	kg/MT	0.0	0.0	0.0	23.4	16.4	12.7
NaHCO3	kg/MT	1.5	1.5	1.5	0.7	1.4	0.9
L-Lysine HCl	kg/MT	4.0	5.0	4.0	2.8	3.1	3.2
DL-Methionine	kg/MT	4.0	4.0	4.0	3.3	2.9	2.5
L-Threonine	kg/MT	1.5	1.5	1.5	0.0	0.0	0.0
Salt	kg/MT	2.0	2.0	2.0	2.9	2.5	2.9
Premix ^1^	kg/MT	4.0	4.0	4.0	5.0	5.0	5.0
Phytase	FYT/kg	1000	1000	1000	1000	1000	1000
AMEn	kcal/kg	2894	2952	3000	2916	3000	3150
Crude protein	%	21.7	20.6	19.2	21.9	19.8	18.5
SID Met + Cys	%	0.95	0.92	0.9	0.91	0.83	0.76
SID Lysine	%	1.21	1.18	1.0	1.21	1.08	1.00
Total Ca	%	0.90	0.89	0.9	1.00	0.79	0.70
Available P	%	0.46	0.42	0.4	0.47	0.36	0.30

^1^ The premix of Trial 1 supplied per kilogram of diet was: vitamin A: 10,000.0 IU; vitamin D3: 2000.0 IU; vitamin E: 30.0 IU; vitamin K3: 2.0 mg; vitamin B1: 1.0 mg; vitamin B2: 5.0 mg; vitamin B6: 3.0 mg; vitamin B12: 12.0 μg; nicotinic acid: 40.0 mg; calcium pantothenate: 10.0 mg; folic acid: 1.0 mg; biotin: 0.1 mg; choline chloride: 400.0 mg; copper: 8.0 mg; iron: 60.0 mg; iodine: 2.0 mg; manganese: 70.0 mg; selenium: 0.15 mg; zinc: 80.0 mg; butylated hydroxytoluene: 4.0 mg; citric acid: 13.8 mg; sodium citrate: 0.4 mg; sepiolite: 0.4 g; calcium carbonate: 2.34 g. The premix of Trial 2 supplied per kilogram of diet was: vitamin A: 12,500.0 IU; vitamin D3: 5000.0 IU; vitamin E: 100.0 IU; vitamin K3: 5.0 mg; vitamin B1: 5.0 mg; vitamin B2: 8.0 mg; vitamin B6: 7.0 mg; vitamin B12: 0.03 μg; nicotinic acid: 100.0 mg; calcium pantothenate: 25.0 mg; folic acid: 3.0 mg; biotin: 0.3 mg; choline chloride: 500.0 mg; copper: 16.0 mg; iron: 50.0 mg; iodine: 1.0 mg; manganese: 80.0 mg; selenium: 0.2 mg; zinc: 80.0 mg.

**Table 2:** Description of qualitative scoring system used in Trial 1 (litter score, gait score, and footpad score) and Trial 2 (footpad score).

Score	Score/Definition (Welfare Quality, 2009)		
	Litter Score	Gait Score	Footpad Score
0	----	The bird walks normally, with no detectable abnormality	No external signs of footpad dermatitis. The skin of the footpad feels soft to the touch and no swelling or necrosis is evident
1	No concretions, litter dry and loose	Slight defect, unduly large strides, slightly uneven gait	The pad feels harder than a non-affected foot. The central part of the pad is raised, reticulate scales are separated, and small black necrotic areas may be present
2	Few and small, discrete concretions, rest of the litter as for score 1	Definite defect in gait but does not hinder the bird from competing for resources (e.g., sufficiently lame on one leg)	Marked swelling of the footpad. Reticulate scales are black, forming scale shaped necrotic areas. The scales around the outside of the black areas may have turned white. The area of necrosis is <1 quarter of the total area of the footpad
3	Cohesive concretions around the drinker and feeder	Obvious gait defect which affects the ability to move about (e.g., limp, jerky, unsteady strut, severe splaying of one leg)	Swelling is evident and the total footpad size is enlarged. Reticulate scales increased in number and separated from each other. The amount of necrosis extends to one half of the footpad
4	Cohesive and compact concretions around the drinker and feeder	Severe gait defect. Still capable of walking when driven or motivated. Squats down at the first opportunity	As for score 3, but with more than half the footpad covered by necrotic cells
5	Cohesive and compact concretions in more than half of the pen	Incapable of sustained walking on its feet. May be able to stand, moving with assistance of the wings or by crawling on the shanks	----

**Table 3:** Effects of a precision biotic (Glycan PB) ingredient ^1^ supplemented at different doses in feed without or with the inclusion of a xylanase enzyme on the growth performance and mortality of broiler chickens raised in floor pens (Trial 1).

Xylanase	Glycan PB Dose	Feed Intake (g/Bird)			BW Gain (g/bird)			cFCR (g Feed/g BW Gain)			Mortality (%)
		0–14 d	0–28 d	0–35 d	0–14 d	0–28 d	0–35 d	0–14 d	0–28 d	0–35 d	0–35 d
No		485	1967	3306	403	1365	1967	1.244	1.486	1.740	2.39
Yes		482	1974	3302	403	1372	1973	1.239	1.479	1.733	2.65
SEM		2.7	8.0	12.3	2.3	6.2	4.8	0.009	0.007	0.006	0.67
	0	491 ^a^	1988	3366 ^a^	396 ^b^	1345 ^b^	1943 ^b^	1.285 ^a^	1.523 ^a^	1.795 ^a^	3.21
	250	476 ^b^	1965	3323 ^b^	407 ^a^	1369 ^a^	1983 ^a^	1.211 ^b^	1.476 ^b^	1.729 ^b^	2.69
	500	484 ^ab^	1960	3222 ^b^	407 ^a^	1391 ^a^	1985 ^a^	1.230 ^b^	1.449 ^c^	1.684 ^c^	1.67
	SEM	3.2	9.6	15.1	2.7	7.4	5.8	0.011	0.008	0.008	0.60
No	0	491	1979	3351	397	1337	1931	1.282	1.448	1.800	2.82
No	250	479	1966	3341	407	1368	1986	1.214	1.451	1.732	3.08
No	500	484	1957	3225	406	1389	1984	1.236	1.472	1.687	1.28
Yes	0	490	1996	3381	394	1352	1954	1.288	1.479	1.790	3.59
Yes	250	473	1963	3306	406	1371	1979	1.207	1.517	1.727	2.31
Yes	500	484	1962	3219	408	1393	1985	1.224	1.529	1.682	2.05
SEM		4.5	13.2	21.3	3.6	11.2	8.2	0.015	0.011	0.011	0.62
Source of variation		------------------------------------Probability ------------------------									
Xylanase		0.52	0.55	0.83	0.86	0.37	0.40	0.71	0.37	0.43	---
Glycan PB		<0.01	0.08	<0.001	<0.01	<0.001	<0.001	<0.001	<0.001	<0.001	---
Xylanase*Glycan PB		0.84	0.74	0.32	0.78	0.78	0.18	0.84	0.89	0.97	---

^a–c^ Means with different superscripts differed at *p* < 0.05. Superscripts are only shown when main effect of treatment had a *p* < 0.05 (*n* = 13 pens/treatment). ^1^ Produced by the catalytic oligomerization of food sugars into tailored glycans [3,4] (Midori USA, Inc., Cambridge, MA, USA, DSM Nutritional Products, Kaiseraugst, Switzerland).

**Table 4:** Effects of a precision biotic (Glycan PB) ingredient ^1^ supplemented at different doses in feed without or with the inclusion of a xylanase enzyme on the litter characteristics, foot pad lesions, and gait scores of broiler chickens raised in floor pens, evaluated at 35 d of age (Trial 1).

Xylanase	Glycan PB Dose	Litter Characteristics							Welfare Assessment			
		Litter pH	Total N (mg/g)	Inorganic N (mg/g)	Ammonia (mg/g)	Ammonia/Inorg. N (mg/mg)	Ammonia/Tot. N (mg/mg)	Litter Score (1–5)	Foot Pad Score 0 (%)	Foot Pad Score 1–4 (%)	Gait Score 0 (%)	Gait Score 1–5 (%)
No		9.12	60.48	19.71	18.00	0.91	0.31	3.18	77.3	22.7	97.8	2.20
Yes		9.31	55.73	20.55	18.70	0.89	0.37	3.13	76.3	23.7	97.5	2.47
SEM		0.10	2.72	1.74	1.63	0.008	0.037	0.08	1.1	1.1	0.6	0.55
	0	9.52	53.80	25.50	23.64	0.92	0.47	3.33 ^a^	72.2	27.8	96.1 ^b^	3.85 ^a^
	250	9.37	62.41	13.33	11.68	0.87	0.19	3.17 ^ab^	75.5	24.5	98.4 ^a^	1.60 ^b^
	500	8.74	58.09	21.56	19.74	0.90	0.35	2.96 ^b^	82.7	17.3	98.4 ^a^	1.56 ^b^
	SEM	0.11	3.33	2.13	1.99	0.010	0.045	0.09	1.4	1.4	0.6	0.62
No	0	9.25 ^bc^	58.70	17.43 ^bc^	15.83 ^cb^	0.95 ^ab^	0.28 ^b^	3.33	70.2 ^c^	29.8 ^c^	96.3	3.71
No	250	9.27 ^abc^	62.20	14.35 ^bc^	12.85 ^cb^	0.93 ^ab^	0.21 ^b^	3.26	77.8 ^abc^	22.2 ^abc^	98.7	1.34
No	500	8.85 ^dc^	60.53	27.35 ^ab^	25.33 ^ab^	0.97 ^a^	0.43 ^ab^	2.95	83.9 ^a^	16.1 ^a^	98.4	1.56
Yes	0	9.80 ^a^	48.90	33.58 ^a^	31.45 ^a^	0.98 ^a^	0.67 ^a^	3.33	74.2 ^bc^	25.8 ^bc^	96.0	4.00
Yes	250	9.48 ^ab^	62.63	12.30 ^c^	10.50 ^c^	0.89 ^b^	0.17 ^b^	3.08	73.2 ^c^	26.8 ^c^	98.1	1.86
Yes	500	8.64 ^d^	55.65	15.78 ^bc^	14.15 ^bc^	0.92 ^ab^	0.26 ^b^	2.97	81.5 ^ab^	18.5 ^ab^	98.4	1.56
SEM		0.15	4.71	3.01	2.82	0.014	0.063	0.13	1.9	1.9	0.8	0.79
Source of variation		----------------------------- Probability ---------------------										
Xylanase		0.08	0.23	0.74	0.76	0.08	0.16	0.62	0.78	0.78	0.69	0.69
Glycan PB		<0.001	0.22	0.002	0.002	<0.001	<0.001	0.05	<0.001	<0.001	0.03	0.03
Xylanase*Glycan PB		0.04	0.57	0.001	0.001	<0.001	<0.001	0.67	0.05	0.05	0.92	0.92

^a–d^ Means with different superscripts differed at *p* < 0.05. Superscripts are only shown when main effect of treatment had a *p* < 0.05. ^1^ Produced by the catalytic oligomerization of food sugars into tailored glycans [3,4] (Midori USA, Inc., Cambridge, MA, USA, DSM Nutritional Products, Kaiseraugst, Switzerland).

**Table 5:** Effects of a precision biotic (Glycan PB) ingredient ^1^ supplemented at 500 g/MT in feed on the growth performance, BW variability, foot pad lesion scores, and mortality of broiler chickens raised in floor cages within a commercial broiler house (Trial 2).

Independent Variable	Negative Control (NC)	NC + Glycan PB	SEM	Probability
Feed intake (g/bird)				
0–10 d	456	508	28	0.19
0–25 d	1890 ^b^	2016 ^a^	42	0.04
0–35 d	2869 ^b^	3034 ^a^	51	0.03
BW Gain (g/bird)				
0–10 d	212	217	2.9	0.28
0–25 d	1164	1187	17	0.33
0–35 d	1882	1994	46	0.10
cFCR (g feed/g BW gain)				
0–10 d	2.248	2.437	0.146	0.37
0–25 d	1.659	1.708	0.035	0.33
0–35 d	1.594	1.547	0.040	0.42
BW CV (%)				
10 d	9.40	9.81	0.50	0.57
25 d	12.1	13.1	0.9	0.43
35 d	13.9	12.6	0.9	0.33
Footpad 35 d				
Score 0 (%)	14.0	27.3	4.6	0.05
Score 1–4 (%)	86.0	72.7	4.6	0.05
Mortality (%)	1.78	3.13	0.96	---

^a,b^ Means with different superscripts differed at *p* < 0.05. Superscripts are only shown when main effect of treatment had a *p* < 0.05. (*n* = 15 pens/treatment). ^1^ Produced by the catalytic oligomerization of food sugars into tailored glycans [3,4] (Midori USA, Inc., Cambridge, MA, USA, DSM Nutritional Products, Kaiseraugst, Switzerland).

## Data Availability

The data that support the findings of this study are available from the corresponding author upon request.
